# Supplementary material for: Luteolin Treatment Ameliorates Brain Development and Behavioral Performance in a Mouse Model of CDKL5 Deficiency Disorder
Source: Int J Mol Sci. 2022 Aug 5;23(15):8719. doi: 10.3390/ijms23158719 (PMC9369425; doi:10.3390/ijms23158719)
Supplement: Supplementary file 1 [file ijms-23-08719-s001.zip › ijms-1825140-supplementary.pdf]

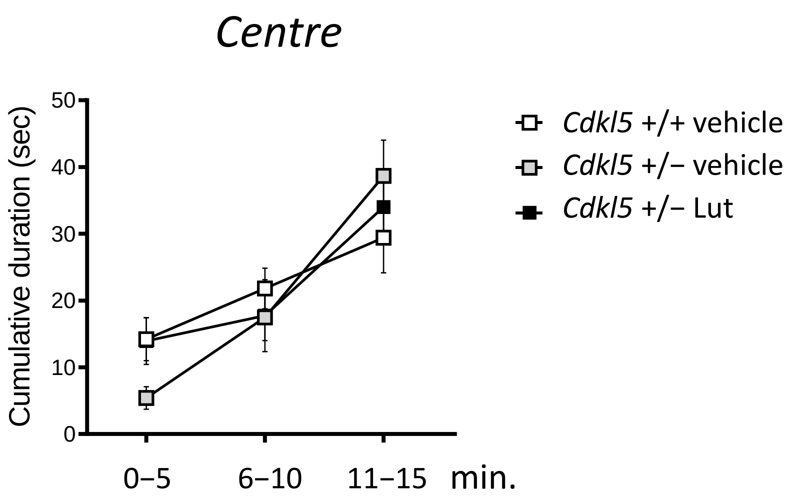

Figure S1. Anxiety-like behavior in *Cdkl*5 +/− mice and effect of luteolin treatment.

Time (cumulative duration) spent in the center of the open-field arena. Vehicle-treated *Cdkl*5 +/+ (n = 19), *Cdkl*5 +/− (n = 18) mice, and luteolin-treated *Cdkl*5 +/− (n = 17) mice spent a comparable time in the center of the arena, suggesting that hyperactivity in *Cdkl*5 +/− was not due to increased anxiety. Fisher's LSD test after two-way ANOVA.

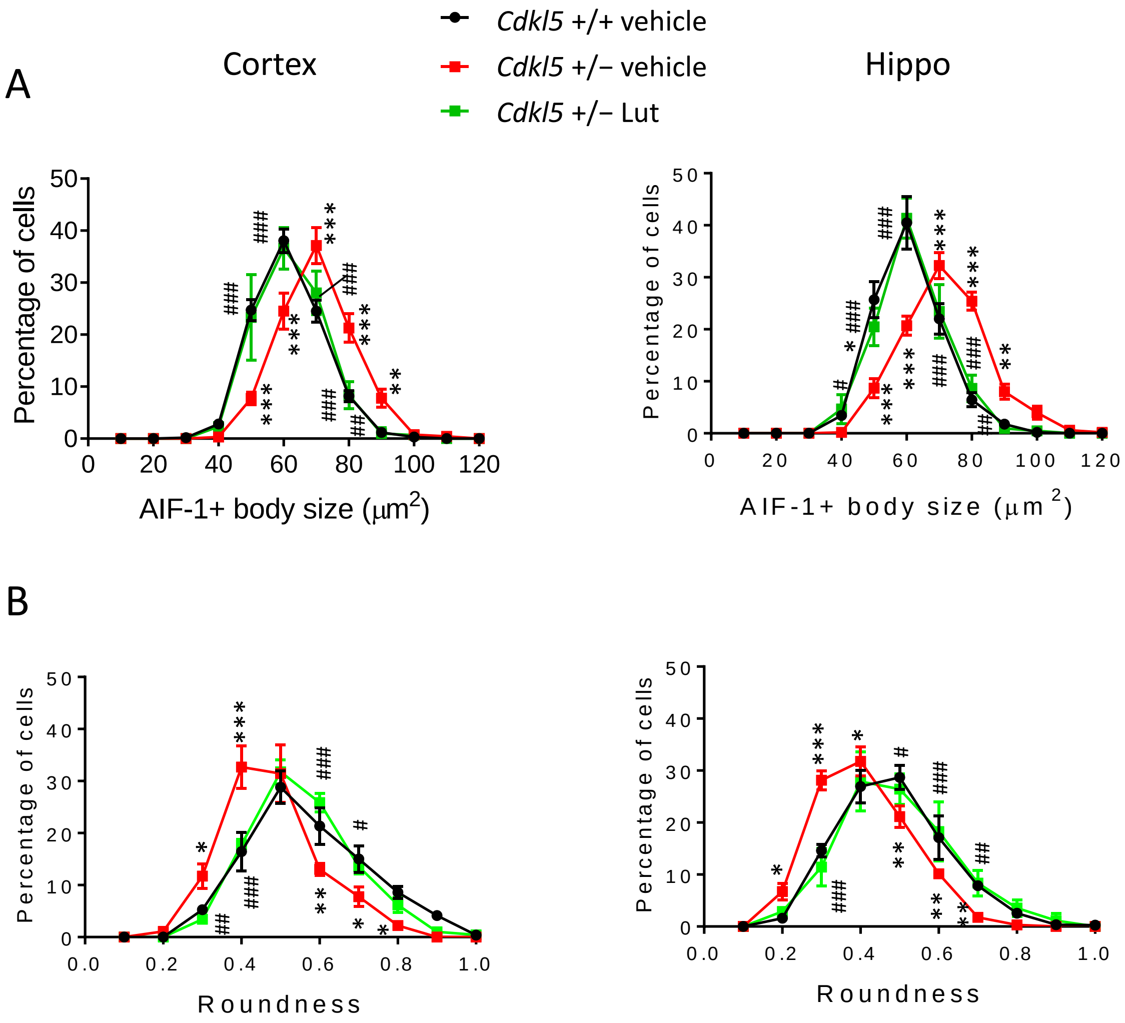

Figure S2. Luteolin treatment completely rescues microglia overactivation in the brain of *Cdkl*5 +/− mice. (A,B) Distribution analysis of microglial cell soma area (A) and microglial cell circularity (roundness; B) in the somatosensory cortex (on the left) and hippocampus (on the right) of vehicle-treated *Cdkl*5 +/+ (n = 5) and *Cdkl*5 +/− (n = 6) mice and luteolin-treated *Cdkl*5 +/− (n = 4) mice. The enlarged body size and irregular shape of microglia cells in the brain of *Cdkl*5 +/− mice was completely restored in 20-day luteolin treated *Cdkl*5 +/− mice. The results in A and B are presented as means ± SEM. *p<0.05, **p<0.01, ***p<0.001 as compared to the vehicle-treated *Cdkl5* +/+ mice; #p<0.01, ##p<0.01, ###p<0.001 as compared to the vehicle-treated *Cdkl5* +/− mice. Fisher's LSD test after two-way ANOVA.

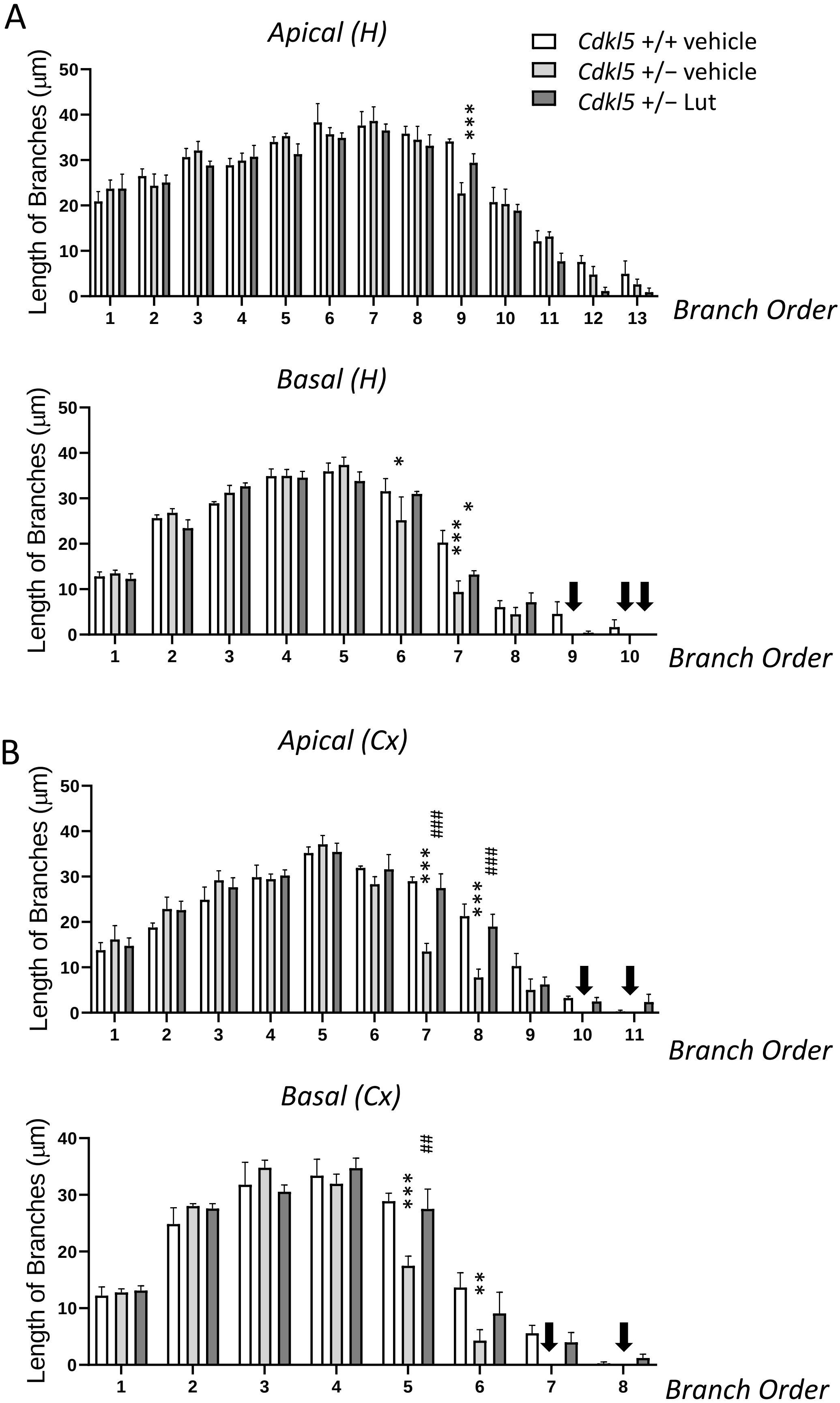
A
Apical (H)
Cdkl5 +/+ vehicle
Cdkl5 +/− vehicle
Cdkl5 +/− Lut
Length of Branches (μm)
Branch Order
Basal (H)
Length of Branches (μm)
Branch Order
B
Apical (Cx)
Length of Branches (μm)
Branch Order
Basal (Cx)
Length of Branches (μm)
Branch Order

Figure S3. Effect of luteolin treatment on mean length of dendritic branches of hippocampal and cortical neurons in *Cdkl*5 +/− mice. (A,B) Quantification of the length of branches of different orders of apical dendrites (upper panel) and basal dendrites (lower panel) in Golgi-stained hippocampal (A) and cortical (B) pyramidal neurons of vehicle-treated *Cdkl5* +/+ (n = 4) and *Cdkl5* +/− (n = 4) mice and luteolin-treated *Cdkl5* +/− (n = 4) mice. Black arrows indicate the lack of branches of that order. H = hippocampus; CX = cortex. Values in A and B are represented as means ± SEM. *p<0.05, **p<0.01, ***p<0.001 as compared to the vehicle-treated *Cdkl*5 +/− condition; ##p<0.01, ###p<0.001 as compared to the vehicle-treated *Cdkl*5 +/− condition. Fisher's LSD test after two-way ANOVA.

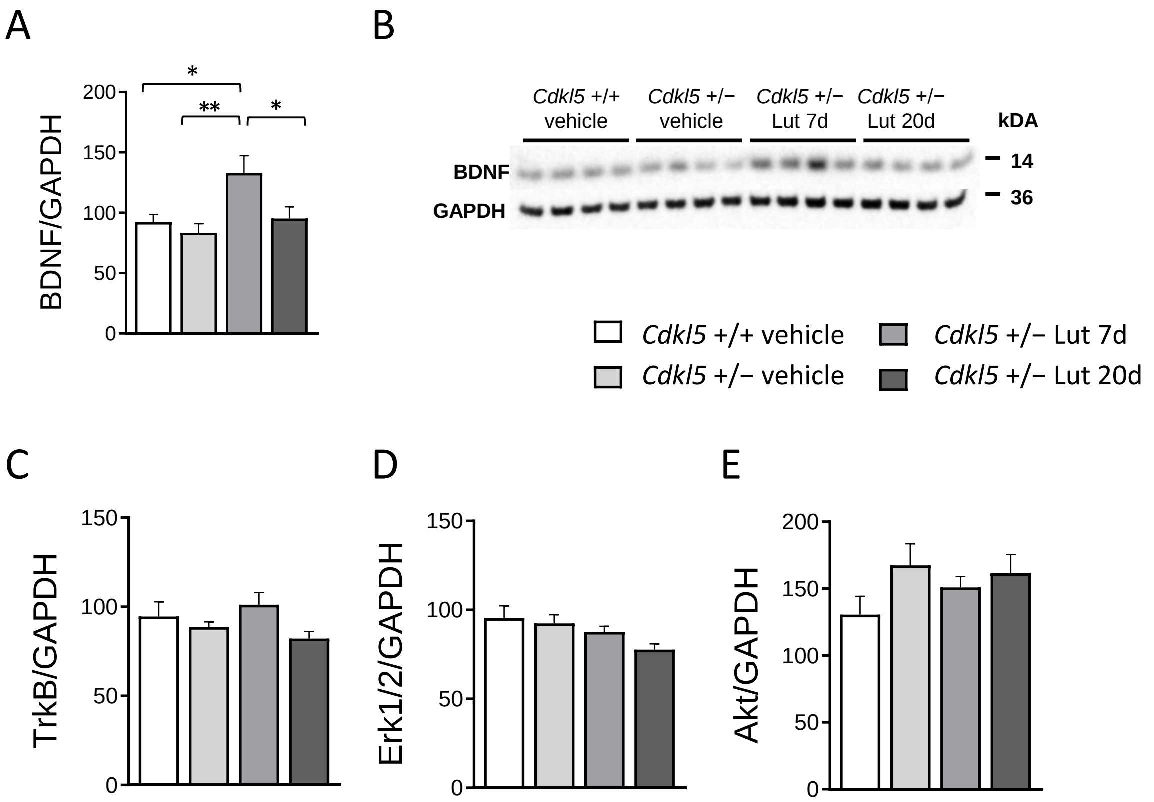

Figure S4. Effect of luteolin treatment on BDNF, TrkB, Erk 1/2 and Akt protein levels in the cortex of *Cdkl*5 +/− mice. (A,B) Western blot analysis of BDNF levels in somatosensory cortex homogenates from vehicle-treated *Cdkl5* +/+ (n = 4) and *Cdkl5* +/− (n = 4) mice, 7-day luteolin treated *Cdkl5* +/− (Lut 7d, n = 4) and 20-day luteolin treated *Cdkl5* +/− (Lut 20d, n = 4) mice. The histogram in A shows mature BDNF protein levels normalized to GAPDH protein levels. **(B)** Example of immunoblot for BDNF; part of the BDNF immunoblot was previously published in [50]. **(C-E)** Histograms show protein levels of TrkB (B), Erk 1/2 (C) and Akt (D) normalized to GAPDH levels in somatosensory cortex homogenates from vehicle-treated *Cdkl5* +/+ (n = 4) and *Cdkl5* +/− (n = 4) mice, 7-day luteolin treated *Cdkl5* +/− (Lut 7d, n = 4) and 20-day luteolin treated *Cdkl5* +/− (Lut 20d, n = 4) mice. Data are expressed as a percentage of vehicle-treated *Cdkl5* +/+ mice. Values represent mean ± SEM. *p<0.05, **p<0.01 (Fisher's LSD test after two-way ANOVA).

					Half emispheres		
**Cohort**	**N° of animals**	**Genotype**	**Treatment**	**Behavioral studies**	**IHC**	**Golgi staining**	**WB**
1	10<br>10<br>10	*Cdkl5* +/+<br>*Cdkl5* +/−<br>*Cdkl5* +/−	Vehicle<br>Vehicle<br>Lut 20	10<br>10<br>10	-<br>-<br>-	-<br>-<br>-	-<br>-<br>-
2	11<br>8<br>8	*Cdkl5* +/+<br>*Cdkl5* +/−<br>*Cdkl5* +/−	Vehicle<br>Vehicle<br>Lut 20	11<br>8<br>8	-<br>-<br>-	-<br>-<br>-	-<br>-<br>-
3	11<br>14<br>14<br>4	*Cdkl5* +/+<br>*Cdkl5* +/−<br>*Cdkl5* +/−<br>*Cdkl5* +/−	Vehicle<br>Vehicle<br>Lut 20<br>Lut 7	-<br>-<br>-<br>-	14<br>20<br>20<br>-	4<br>4<br>4<br>-	4<br>4<br>4<br>4

Table S1. List of cohorts of treated mice and their use. WB: western blotting, IHC: immunohistochemistry.

	Target	Description	Dilution	Manufacturer
**Primary Antibodies**	AIF-1	Rabbit polyclonal	IHC 1:300	Thermo Fisher Scientific
	Ki67	Rabbit monoclonal	IHC 1:200	Thermo Fisher Scientific
	DCX	Goat polyclonal	IHC1:100	Santa Cruz Biotechnology
	DCX	Rabbit polyclonal	IHC1:300	Thermo Fisher Scientific
	BrdU	Rat monoclonal	IHC 1:200	Abcam
	PSD95	Rabbit polyclonal	IHC 1:200	Abcam
	GAPDH	Rabbit polyclonal	WB 1:5000	Sigma-Aldrich
	BDNF	Rabbit polyclonal	WB 1:500	Santa Cruz
	P-TrkB (Ser816)	Rabbit polyclonal	WB 1:500	Millipore
	TrkB	Rabbit polyclonal	WB 1:500	Santa Cruz
	P-Erk1/2 (Thr202/Tyr204)	Rabbit polyclonal	WB 1:1000	Cell signaling
	Erk1/2	Rabbit polyclonal	WB 1:1000	Cell signaling
	P-Akt (Ser473)	Rabbit polyclonal	WB 1:1000	Cell signaling
	Akt	Rabbit polyclonal	WB 1:1000	Cell signaling
**Secondary Antibodies**	Goat anti-Rabbit IgG HRP-conjugated		WB 1:5000	Jackson ImmunoResearch
	Biotinylated anti-goat IgG		IHC 1:200	Vector Bio-Labs
	Goat anti-mouse IgG Cy3-conjugated		IHC 1:200	Jackson ImmunoResearch
	Goat anti-rat IgG Cy3-conjugated		IHC 1:200	Jackson ImmunoResearch
	Goat anti-rabbit IgG Cy3-conjugated		IHC 1:200	Jackson ImmunoResearch

Table S2. List of primary and secondary antibodies. WB: western blotting, IHC: immunohistochemistry.

	Before treatment	End of the treatment	
	**Vehicle**	**Vehicle**	p
***Cdkl5* +/+**	20.37 ± 0.32	20.36 ± 0.30	n.s.
	**Vehicle**	**Vehicle**	
***Cdkl5* +/−**	20.72 ± 0.44	20.87 ± 0.37	n.s.
p	n.s.	n.s.	
	**Luteolin**	**Luteolin**	
***Cdkl5* +/−**	20.23 ± 0.62	20.82 ± 0.48	n.s.
p	n.s	n.s	

Table S3. Effect of treatment with luteolin on body weight. Body weight in grams of *Cdkl5* +/+ (vehicle n = 21) and *Cdkl5* +/− (vehicle n = 18 or luteolin n = 18) mice at the beginning and at the end of a 20 days of daily treatment with vehicle or luteolin (10 mg/kg). Values are represented as mean ± SEM, n.s. not significant (two-tailed Student’s t-test).
